# Emission Characteristics, Co-Drivers, and Mitigation Implications of NH_3_, N_2_O, and CH_4_ from Livestock Manure in China from 2013 to 2023

**DOI:** 10.3390/toxics13110933

**Published:** 2025-10-30

**Authors:** Xiaotang Zhang, Zeyan Wu, Junchi Wang, Qinge Sha

**Affiliations:** 1 College of Environment and Energy, South China University of Technology, Guangzhou 510006, China; zhangxiaotang2022@163.com; 2 College of Environment and Climate, Jinan University, Guangzhou 511436, China; moose1111@163.com; 3 College of Ecology, Hainan University, Haikou 570228, China; wangjunchi987@163.com

**Keywords:** livestock manure, China, emission trends, carbon neutrality

## Abstract

Livestock and poultry manure emits substantial amounts of ammonia and non-CO_2_ greenhouse gases of nitrous oxide and methane, contributing simultaneously to climate forcing and air quality degradation. However, few studies have provided an integrated quantification of ammonia, nitrous oxide and methane emissions across multiple species and provinces in China. This study established a coupled provincial inventory for 2013–2023 and applied the Logarithmic Mean Divisia Index (LMDI) to identify socioeconomic drivers. Results show that NH_3_ emissions declined slightly from ~4.1 Tg in 2013 to 3.95 Tg in 2023 (−3.7%), while N_2_O increased from 2.1 to 2.3 Tg (+9.5%) and CH_4_ rose from 3.1 to 4.2 Tg (+35%). Consequently, the aggregated global warming potential increased by ~24% (from ~1100 to ~1370 Tg CO_2_-eq). Hogs were identified as the dominant contributor across gases. High-emission provinces contributed disproportionately, whereas metropolitan and western provinces reported marginal levels. LMDI decomposition revealed that affluence and technological intensification were the main drivers of growth, partially offset by production efficiency and labor decline. This study provides one of the first integrated multi-gas, multi-species, and region-specific assessments of livestock manure emissions in China, offering insights into targeted mitigation strategies that simultaneously support carbon neutrality and air quality improvement.

## 1. Introduction

China is an agriculture-based country, with rising affluence and a stable population increasing consumption demand for animal-based foods [1]; the scale of the populations of livestock and poultry farming has been continuously expanding [2]. According to the China Statistical Yearbook [3], the populations of cattle, goats, and poultry increased by 48%, 79%, and 49%, respectively, between 2010 and 2020. Concurrently, the economic output of the livestock sector has grown substantially, with livestock GDP rising from CNY 1331 billion in 2000 to 2058 billion in 2020 [4]. Its share of total agricultural gross domestic product (GDP) increased from 12.4% to 35.5% over the same period [5]. Livestock production has become a pillar industry within China’s agricultural and rural economy. The expansion of livestock numbers and GDP has led to a corresponding increase in manure production [6]. Over the past decade, China’s livestock sector produced approximately 3.5 to 3.8 billion tons of manure annually [7]. Although the comprehensive utilization rate of livestock manure has reached 60–75%, a substantial amount remains untreated, causing serious environmental pollution and climate change [8].

Livestock manure simultaneously emits NH_3_, N_2_O, and CH_4_ [9], increasing global warming potential (GWP) and posing combined air quality and climate impacts. Fecal organic matter and nitrogenous compounds are hydrolyzed by urease to produce ammonium (NH_4_^+^), which can volatilize into the atmosphere as gaseous NH_3_ [10]. During nitrification, aerobic nitrifying bacteria oxidize NH_4_^+^ to nitrite (NO_2_^−^) and subsequently to nitrate (NO_3_^−^), with small amounts of N_2_O emitted as a by-product [11]. Denitrifying microbes also produce N_2_O during the stepwise reduction of NO_3_^−^/NO_2_^−^ to N_2_ under oxygen-limited conditions. In anaerobic conditions, the anaerobic bacteria will decompose organic matter and methanogens are converted to CH_4_ [12]. Based on simulations, the radiative efficiencies of CH_4_ and N_2_O are 3.63 × 10^−4^ and 2.987 × 10^−3^ W m^−2^ ppb^−1^, respectively, while that of CO_2_ is 1.37 × 10^−5^ W m^−2^ ppb^−1^. Accordingly, the global warming potentials (GWP100) of CH_4_ and N_2_O are 28 and 273 times higher than that of CO_2_, respectively. In China, livestock production emits approximately 4000–5500 kt of NH_3_ [13,14,15], 44 kt of N_2_O [16,17,18], and 867–1000 kt of CH_4_ annually [19,20,21]. It is estimated that livestock manure contributes approximately 52%, 29%, and 11% of agriculturally derived NH_3_, N_2_O and CH_4_ emissions in China, respectively. China officially announced the national strategic goal of achieving carbon neutrality by 2060 [22], aiming to attain net-zero greenhouse gas (GHG) emissions. In the context of carbon neutrality and air quality improvement, it is essential to consecutively assess the magnitude of livestock manure emissions and identify mitigation opportunities as the mitigation targets are scheduled [23].

A comprehensive assessment of the emission characteristics, underlying co-drivers, and policy implications of livestock farming air pollutants—NH_3_, the non-CO_2_ greenhouse gas N_2_O, and CH_4_—is critical for designing targeted mitigation strategies and for projecting future environmental and climate impacts [24,25,26]. Previous research efforts at global or national scales have sought to identify, characterize, and estimate livestock emissions from diverse sources concentrated on single gases. For example, Huang et al. [13] estimated that ammonia (NH_3_) emissions from livestock in 2006 were 5.3 Tg, accounting for approximately 54% of the national total. Henan, Hebei, and Shandong were the highest-emitting provinces, whereas Hong Kong and Macao showed zero emissions. By category, cattle were the largest source (1.9 Tg NH_3_ yr^−1^), followed by laying hens and pigs (0.7 Tg NH_3_ yr^−1^ each). Kang et al. [14] estimated NH_3_ emissions from animal husbandry and characterized their spatiotemporal distribution, showing an increase from 2.8 Tg (1980) to 5.1 Tg (2012) and hotspot concentrations in eastern China, eastern Sichuan, and portions of Xinjiang. Xu et al. [27] used county-level statistics for 1978–2008 to estimate NH_3_ emissions from the land application of livestock manure and to assess mitigation potential via scenario analysis. Emissions from manure spreading were 3.8 Tg, with source shares of cattle (30.2%), pigs (28.9%), poultry (26.2%), and dairy cattle (7.9%). Zhang et al. [15] recompiled the 2008 agricultural ammonia emission inventory of China using a “bottom-up” emission coefficient method combined with a “top-down” remote sensing inversion method, quantified NH_3_ emissions of 5.31 Tg from agriculture livestock waste in China for the year 2008, and identified a pronounced summer peak driven by temperature.

Recent works have advanced understanding of the emission of non-CO_2_ greenhouse gases such as CH_4_ and N_2_O from livestock manure. For instance, Liang et al. [28] compiled a full-scale national inventory spanning four decades from 1980 to 2020, showing N_2_O emissions rising from 79.9 Gg in 1980 to 119.3 Gg in 2000, then declining to 91.7 Gg in 2020, with livestock manure contributing 26.3% of total N_2_O emissions. Complementing this, Luo et al. [29] analyzed temporal and spatial patterns from 1978 to 2015, reporting that livestock-related N_2_O emissions nearly doubled up to the early 2000s (about 20% share), then gradually decreased with a turning point around 2004, trends they linked to intensification, herd scaling, and evolving husbandry techniques. In the multigap context, Yuan et al. [30] quantified China’s greenhouse gas budget for 2000 to 2023, highlighting the roles of manure management in N_2_O (13.1%) and CH_4_ (5.0%) source contributions. Looking forward, Chen et al. [31] assessed mitigation trajectories for non-CO_2_ greenhouse gases in Chinese agriculture and identified livestock management, including improved manure handling and reduced enteric fermentation via feed strategies, as providing the largest technical reduction potential (46%). However, previous emission inventories of N_2_O and CH_4_ have relied mostly on European observational and experimental data. Therefore, it is important that N_2_O and CH_4_ emission values are re-evaluated by considering regional characteristics. On the other hand, direct comparisons of NH_3_, N_2_O, and CH_4_ gas emission characteristics from the manure of different livestock species remain scarce in the literature. Furthermore, there is insufficient clarity on which regions and livestock types should be targeted and prioritized to effectively advance mitigation efforts.

To address these challenges, this study refines China’s livestock manure emission estimates using species-specific, region-specific data and develops a coupled, integrated inventory that simultaneously quantifies air pollutants of NH_3_ and greenhouse gases of N_2_O and CH_4_, replacing the conventional practice of constructing separate air pollutants and greenhouse gas inventories. Based on a long-term historical inventory of livestock, we analyzed changes in emissions, variations in source contributions, and spatial patterns. Meanwhile, we used the Logarithmic Mean Divisia Index (LMDI) method to reveal the socioeconomic drivers of livestock emissions. Further, the global warming potential (GWP) associated with manure from different livestock species was simultaneously analyzed and compared across China’s provinces. These findings offer a scientific and data-driven basis to support the implementation of China’s “Carbon Neutrality Strategy”. This study also offers valuable insights into regional ecological security and sustainable green agriculture.

## 2. Methods and Datasets

### 2.1. Study Domain and Source Categories

Given pronounced differences across China’s regions and provinces in climate conditions, livestock composition, and production practices, this study divided the area into six macro-regions, according to the administrative division codes of the People’s Republic of China [32] and the Resource and Environment Data Cloud Platform [33] to objectively assess regional and inter-provincial disparities in agricultural livestock emissions. Therefore, this study encompasses 31 provinces, municipalities, and autonomous regions in mainland China; Taiwan, Hong Kong, and Macao were excluded due to data limitations.

**Figure 1:**
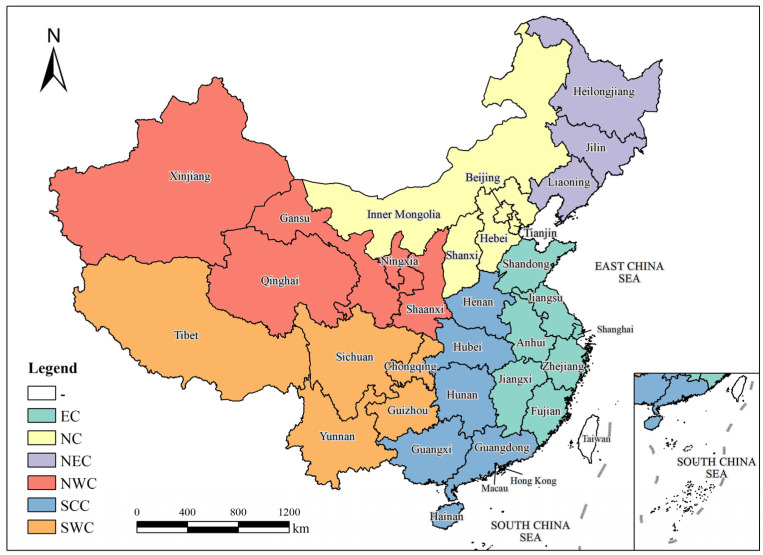
Study areas.

As shown in Figure 1, the agricultural regions were divided as follows: (i) Northeast China (NEC: Liaoning, Jilin, and Heilongjiang), dominated by agro-livestock systems in major grain production areas with strong crop–livestock integration; (ii) North China (NC: Beijing, Tianjin, Shanxi, Hebei, and Inner Mongolia), characterized primarily by grassland pastoral systems and supplemented by suburban intensive livestock production; (iii) East China (EC: Shanghai, Jiangsu, Zhejiang, Anhui, Fujian, Jiangxi, and Shandong), dominated by agricultural livestock systems; (iv) South Central China (SCC: Henan, Hubei, Hunan, Guangdong, Guangxi, and Hainan), also dominated by agricultural livestock systems with high pig and poultry densities; (v) Southwest China (SWC: Chongqing, Sichuan, Guizhou, Yunnan, and Tibet), characterized by mountainous livestock production and highland pastoral systems; and (vi) Northwest China (NWC: Shaanxi, Gansu, Qinghai, Ningxia, and Xinjiang), representing vast grassland and desert pastoral systems. This study encompasses 31 provinces, municipalities, and autonomous regions in mainland China. Taiwan was excluded due to the lack of accessible agricultural statistical data, while Hong Kong and Macau were excluded because of their limited agricultural activities and data unavailability.

Based on the classification system of the China Agricultural Statistical Yearbook and our previous work [34], the emission sources in this inventory were updated to include 17 categories in total, comprising 10 livestock categories and 7 poultry categories (Table 1). To maintain methodological and pollutant coherence with the livestock manure management framework, CH_4_ from enteric fermentation was not considered. Detailed activity data for different livestock and poultry species numbers are listed in Supplementary Materials Table S1.

### 2.2. Calculation of Livestock Emissions

This study develops a bottom-up approach that uses consistent province-level activity data and species-specific emission factors to estimate emissions and then aggregates the results to the national level. Using this framework, we calculate NH_3_, N_2_O, and CH_4_ emissions from China’s livestock sector for 2013–2023, as shown below:

(1) $$E_{i}=\sum_{j}\left({AD}_{i,j,m}\times EF_{i,j,m}\right)$$

where *Ei* denotes the total estimated emissions for the source category; *i*, *j*, and *m* represent the source type, the province in China, and the month, respectively; *AD_i_*_,*j*,*m*_ refers to the activity level associated with the category; and EF*_i_*_,*j*,*m*_ is the corresponding specific condition emission factor for the category. The detailed calculation methods for compiling activity data and emission factors for each source were presented and discussed by Zhang et al. [34].

#### 2.2.1. Calculation of NH_3_ Emissions

Livestock-related NH_3_ emissions were estimated following the methodology established by Zheng et al. [24], which provides a systematic framework for compiling agricultural emission inventories. NH_3_ emissions from livestock manure management were calculated as follows:

(2) $$E_{LMM,i}=\sum_{jk}{PL}_{j,k}\times {EF}_{LMM,i,k,l,t}$$

where *E_LMM_*_,*i*_ denotes the NH_3_ emissions from *LMM*, *i* represents NH_3_, and *PL_j_*_,*k*,*l*_ (head) represents the population of livestock for *j* province, *k* source of livestock, and *l* type of breeding method. *EF_i_*_,*j*,*k*,*l*,_*_t_* are the emission factors for *i* gas, *k* sub-source of livestock, *l* type of breeding method (kg/head), and *t* temperature.

Emission factors were modified based on temperature-dependent parameters following the *Technical Guidelines* [36]. Pixel-level monthly EFs were obtained by adjusting nitrogen excretion rates, feeding times, and NH_3_ volatilization rates across different livestock categories. NH_3_ emissions from livestock were estimated across four manure management stages, housing, storage, land spreading, and grazing, using the following equations:

Housing (ef_1_):

ef_1_ = *N*_X1_ *V*_1_ (3)

Storage (ef_2_):

ef_2_ = *N*_X1_ (1 − *V*_1_) *V*_2_ (4)

Land Spreading (ef_3_):

*e*f_3_ = *N*_X1_(1 − *V*_1_) (1 − *V*_2_) *V*_3_ (5)

Grazing (ef_4_):

ef_4_ = N_X4_ V_4_ (6)

Total Emission Factor (EF):

*EF* = ef_1_ + ef_2_ + ef_3_ + ef_4_ (7)

where ef_1,2,3,4_ are the NH_3_ emissions at different stages. *N_X_*_1,4_ are nitrogen excretions during housing (1) and grazing (4), respectively. *V*_1,2,3,4_ are the temperature-dependent NH_3_ volatilization rates at each stage, and EF is the final emission factor. Detailed NH_3_ emission factors for different livestock and poultry species under different temperatures are listed in Supplementary Materials Table S2.

#### 2.2.2. Calculation of N_2_O Emissions

We applied emission inventory methodologies from the province’s Guidelines for the National Emission Inventory [37]. Direct N_2_O emissions from livestock manure arise from two main processes: (*i*) nitrification and denitrification of nitrogen in manure during storage and treatment, and (j) subsequent emissions following the application of manure to soils. Detailed emission factors are provided in Supplementary Table S3. The first summation term represents N_2_O emissions during manure storage and processing, while the second term accounts for emissions released after manure application to agricultural soils.

(8) $$E_{N_{2}O}=\sum_{i}(N_{ex}(i)\times MS(i)\times EF_{N_{2}O}(i))+\sum_{j}(N_{app}(j)\times EF_{N_{2}O-soil}(j))$$

where $E_{N_{2}O}\;$ is total direct N_2_O emissions (kg N_2_O yr^−1^), *N_ex_*(*i*) is the amount of nitrogen excreted by livestock category *i* (kg N yr^−1^), *MS*(*i*) is the fraction of manure managed under the system, $EF_{N_{2}O}(i)$ is the emission factor for direct N_2_O emissions from manure management system *i* (kg N_2_O–N per kg N excreted), *N_app_*(*j*) is the amount of nitrogen applied to soils from manure management system *j* (kg yr^−1^), and E$F_{N_{2}O-soil}(j)$ is the emission factor for direct N_2_O emissions from manure applied to soils (kg N_2_O–N per kg N applied).

#### 2.2.3. Calculation of CH_4_ Emissions

We applied emission inventory methodologies from the province’s Guidelines for the National Emission Inventory [37]. This study did not include intestinal fermentation of livestock and poultry, as shown below:

(9) $$E_{i}=\sum_{j}({AD}_{i,j,m}\times EF_{i,j,m})$$

where E_i_ denotes the total estimated emissions for the source category; *i*, *j*, and *m* represent the source type, the province in China, and the month, respectively; AD*_i_*_,*j*,*m*_ refers to the activity level associated with the category; and EF*_i_*_,*j*,*m*_ is the emission factor for the category (Table S4).

#### 2.2.4. Calculation of GWP Emissions

IPCC has used the global warming potential (GWP) to allow for comparisons of the global warming impacts of different gases [38]. Specifically, the GWP is a measure of how much energy the emission of 1 ton of a gas will absorb over a given period of time, relative to the emission of 1 ton of carbon dioxide (CO_2_). The larger the GWP, the more a given gas warms the Earth compared to CO_2_ over that time period. The time period usually used for GWPs is 100 years. GWPs provide a common unit of measure, which allows analysts to add up emissions estimates of different gases:

GWP = 28 × E_CH4_ + 256 × (E_N2O_ + 0.01E_NH3-N_ × 44/28) (10)

where GWP is the greenhouse gas global warming potential, kt, expressed in CO_2-_eq; E_CH4_ is the cumulative CH_4_ emissions, kt^−1^; E_N2O_ is the cumulative N_2_O emissions, kt^−1^; and E_NH3-N_ is the cumulative NH_3_-N emissions, kt^−1^. Additionally, 28 and 265 are the 100-year warming potentials of CH_4_ and N_2_O relative to CO_2_, respectively (IPCC, 2013); 0.01 is the conversion coefficient of NH_3_ (measured as NH_3_-N) to N_2_O (measured as N_2_O-N) through atmospheric deposition and chemical reactions; and 44/28 is the conversion coefficient of N_2_O-N to N_2_O.

### 2.3. Spatial Allocation of Livestock Emissions

Emissions from livestock manure management (LMM) were allocated based on the spatial distribution of rural residential areas. Livestock emissions were spatially allocated to 9 km × 9 km grid cells using a Geographic Information System (ArcGIS, version 10.2). The allocation was performed according to the following equation:

(11) $$E_{i,j}=E_{total}\times (\frac{W_{i,j}}{{\sum i,jW}_{i,j}})$$

where E*_i_*_,*j*_ presents the emissions allocated to grid cell (*i*,*j*), E*_total_* is total livestock emissions for the region. *W_i_*_,*j*_ is the weighting factor for grid cell (*i*,*j*) based on agricultural activity, and *∑_i_*_,*j*_ W*_i_*_,*j*_ is the sum of weighting factors across all grid cells.

### 2.4. Uncertainty Analysis

Various sources of uncertainty in emission factors, activity data, and calculation parameters [39] can lead to uncertainties in emission estimates. In this study, a quantitative approach using the Monte Carlo model with R (version 4.2.3) was applied to evaluate the uncertainty of all sources of livestock emissions according to the method by Zheng et al. [24]. By establishing emission factor databases from the literature, the uncertainties for each source and total emissions were quantified and key sources leading to uncertainty in model outputs were identified using sensitivity analysis approaches. Detailed input parameters are provided in Supplementary Table S5.

### 2.5. Driving Force Analysis Based on the LMDI

To investigate the determinants of livestock emissions, this study employed the Logarithmic Mean Divisia Index (LMDI) decomposition approach, which has been widely used in emission factor analysis owing to its additive property, absence of residuals, and consistency across decomposition forms [40]. The general identity can be expressed as follows:

(12) $$E=\frac{E}{S}\times \frac{S}{A}\times \frac{A}{M}\times \frac{M}{L}\times \frac{L}{P}\times P\;=\sum_{i}∆\alpha \;\times ∆\beta \times ∆\gamma \times ∆\rho \times ∆\delta \;\alpha =E/S,\;\beta =S/A,\;\gamma =A/M,\;\rho =M/L,\;\delta =L/P$$

where α is agricultural livestock production efficiency, β is agricultural industrial structure, γ is agricultural affluence, ρ is agricultural technology level, and δ is agricultural population labor force. The 5 components are explained in Table 2.

## 3. Results and Discussion

### 3.1. Emissions Trends and Comparison with Previous Studies

**Figure 2:**
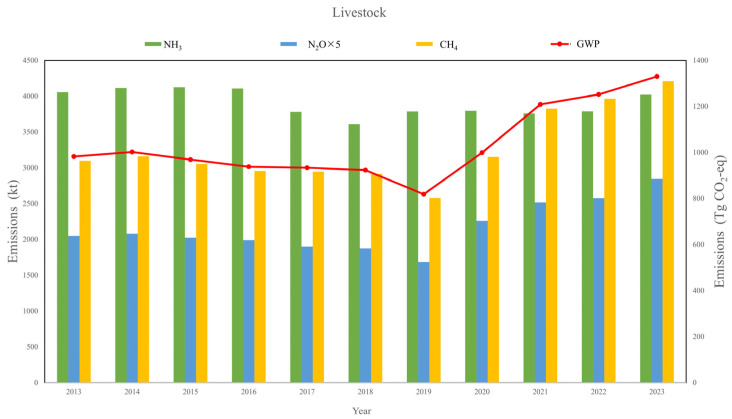
Historical livestock emissions from 2013 to 2023 in China.

Livestock-related NH_3_, N_2_O, and CH_4_ emissions in China exhibited divergent trajectories during 2013–2023 (Figure 2). NH_3_ emissions decreased slightly from approximately 4.1 Tg in 2013 to 3.95 Tg in 2023 (−3.7%), reflecting improvements in manure management and partial adoption of emission mitigation practices. In contrast, N_2_O emissions increased from ~2.1 Tg to 2.3 Tg (+9.5%), driven largely by manure storage and treatment processes. CH_4_ emissions rose more substantially, from ~3.1 Tg in 2013 to ~4.2 Tg in 2023 (+35%), consistent with the expansion of ruminant livestock populations. As a result, the aggregated GWP of livestock emissions increased steadily from ~1100 Tg CO_2_-eq to ~1370 Tg CO_2_-eq (+24%), highlighting the dominant climate forcing role of CH_4_. These results align with previous studies that reported sustained growth in livestock-related non-CO_2_ GHG emissions in China. Our estimates of NH_3_ are broadly consistent with national inventories, while the upward CH_4_ trend parallels findings from the Emissions Database for Global Atmospheric Research (EDGAR) [41] and the Greenhouse Gas and Air Pollution Interactions and Synergies (GAINS) databases [42], though with slightly higher values, likely due to updated emission factors and refined activity data.

### 3.2. Contribution by Livestock Emissions

**Figure 3:**
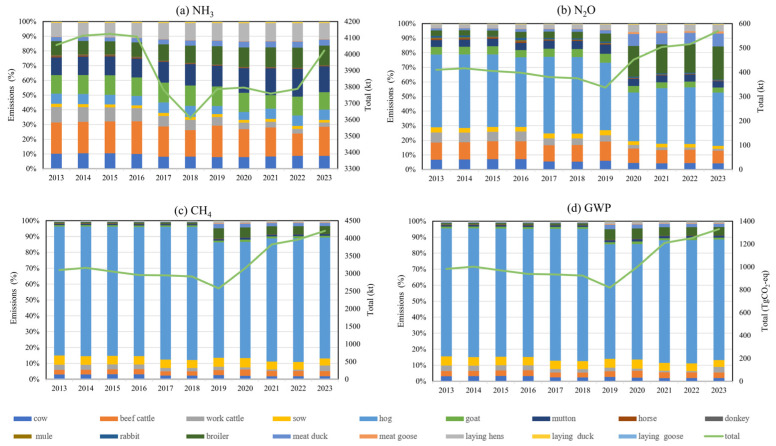
Contributions of livestock types to total NH_3_, N_2_O, and CH_4_ emissions from 2013 to 2023 in China.

Figure 3 presents the relative contributions of different livestock categories specific to total agricultural livestock emissions of NH_3_, N_2_O, CH_4_, and the aggregated global warming potential (GWP) in China during 2013–2023. Distinct emission patterns are observed across gas species. For NH_3_ and N_2_O (Figure 3a,b), emissions are primarily attributable to pigs (sows and hogs), followed by cattle (dairy and beef). Poultry (broilers, layers, ducks, and geese) and small ruminants (sheep and goats) provide comparatively smaller contributions, though their shares exhibit a slight upward trend after 2018.

In addition, the contribution of the livestock manure management N_2_O is depicted in Figure 3b. It demonstrates that pigs, cattle, and poultry produce significant amounts of emissions, accounting for 40.97%, 19%, and 13% of total livestock N_2_O emissions in 2023. This could be ascribed to the relatively quick increase in meat, egg, and milk consumption in recent years, which resulted in greater production of these animals to meet the population’s daily nutrition requirements. Hog emissions constituted the largest proportion of the livestock emissions, which is related to people’s dietary habits. Emissions from poultry now make a more significant contribution due to the sharply increased consumption in recent years. In contrast, the lesser contributions provided by work livestock (30% to 5%) (include the work cattle, horses, donkeys, and mules) were discovered to be related to lower levels of activity data that were predominantly obtained for draft animals.

Figure 3c illustrates the livestock manure management CH_4_ emissions of the major categories. The results show that cattle’s relative contributions to these components have increased while work livestock emissions have dramatically decreased. With 4.5% of CH_4_ emissions coming from draft animals, they were the primary cause of emissions in 2000, whereas cattle contributed 4.6% of CH_4_ emissions, being the largest source of emissions in 2023. Pigs and sheep contributed significantly to the overall emissions at this time as well. The change in the proportion of contributing sources is caused by the increase in the number of beef cattle breeding, which suggests that more effective emission reduction measures should be taken at this source.

The relative contributions of different livestock types to total NH_3_, N_2_O, and CH_4_ emissions in China from 2013 to 2023 reveal clear species-specific patterns (Figure 3). NH_3_ emissions were predominantly associated with beef cattle production, accounting for nearly half of the total throughout the study period, followed by cattle and poultry, which together contributed more than 30%. This finding is consistent with previous studies that identified hog manure management as the single largest source of agricultural NH_3_ emissions in China [27]. N_2_O and CH_4_ emissions showed a similar structure, with hogs as the major contributor and poultry providing a smaller but gradually increasing share, in agreement with reports linking intensive poultry and swine housing to rising N_2_O release from manure storage. Consequently, the aggregated GWP of livestock emissions was primarily determined by hog CH_4_ emissions, although hog-derived NH_3_ and N_2_O also exerted a notable influence. Overall, these stable yet differentiated contribution patterns underscore the necessity of targeted mitigation measures, such as improving manure management in hog and poultry systems.

### 3.3. Spatial Variations

**Figure 4:**
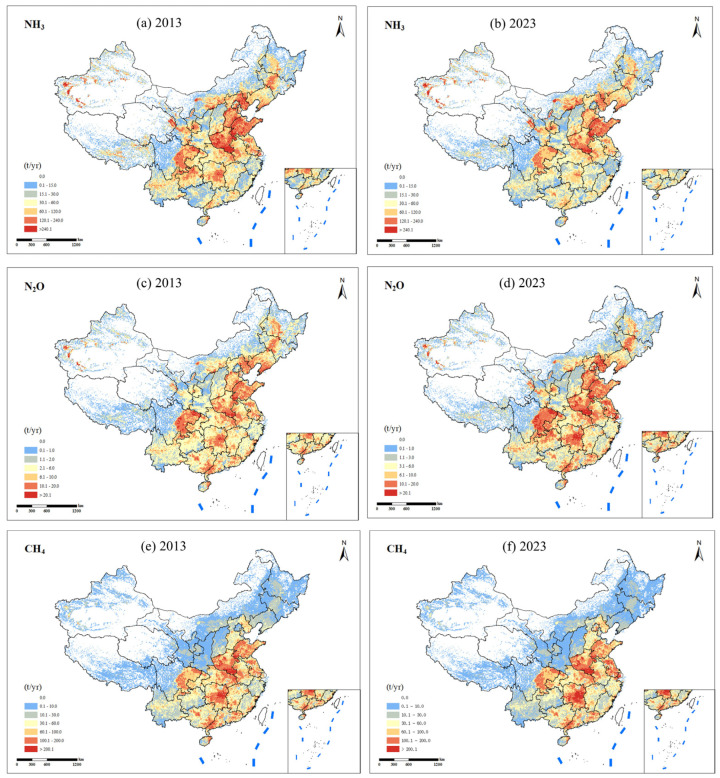
Spatial distributions of NH_3_, N_2_O, and CH_4_ emissions in livestock for 2013 and 2023 in China (**a**) 2013 NH_3_, (**b**) 2023 NH_3_, (**c**) 2013 N_2_O, (**d**) 2023 N_2_O, (**e**) 2013 CH_4_, and (**f**) 2023 CH_4_.

Spatial patterns of livestock-related NH_3_, N_2_O, and CH_4_ emissions in China exhibit pronounced regional heterogeneity (Figure 4). A comparison between 2013 and 2023 highlights both persistence and shifts in major emission hotspots. For NH_3_, the North China Plain and the Sichuan Basin remain dominant high-emission zones; however, emission intensity has slightly declined in parts of Shandong and Hebei while expanding southward into Hunan and Jiangxi, reflecting regional adjustments in livestock production and manure management practices. N_2_O emissions display similar spatial persistence but with intensified hotspots in Heilongjiang and Xinjiang, consistent with the expansion of large-scale livestock operations and forage crop cultivation. CH_4_ emissions remain concentrated in the Northeast, North China Plain, and Sichuan Basin, yet the spatial extent of high-emission areas has expanded notably in Heilongjiang and Inner Mongolia, indicating intensification of ruminant livestock systems.

These redistributions are attributable to three mechanisms, primarily structural adjustment and partial relocation of production toward central and western provinces. Additionally, tighter coupling of herds to local feed and grassland resources in the Northeast and Northwest due to climate change is increasing precipitation and temperature in the north. Finally, during the COVID-19 pandemic, migrant workers moved from eastern cities to rural areas, increasing the economic income through livestock and poultry farming, leading to increased emissions. Overall, while the primary emission clusters have persisted, the observed redistribution and intensification trends underscore the need for mitigation strategies that increasingly address emerging hotspots in central and western provinces alongside the traditionally dominant regions.

### 3.4. Identification of Key Uncertainty Sources

A comprehensive uncertainty analysis was conducted to evaluate the robustness of estimates of NH_3_ and GHG (N_2_O and CH_4_) emissions originating from livestock manure management (LMM) sources (Table 3). The uncertainty of NH_3_, N_2_O, and CH_4_ was estimated to be (−70.51%; 112.77%), (−52.34%; 71.63%), and (−67%; 136.07%), respectively. The uncertainty for livestock emissions is relatively moderate in this study because we applied a bottom-up approach and adopted recalibrated, China-specific emission factors with parameter adjustments for Chinese conditions, rather than relying on unmodified factors from other regions. This localization of parameters improves consistency with observed practices and helps constrain the uncertainty. Meanwhile, the results demonstrate pronounced variability across various agricultural sub-sectors for livestock. Poultry emissions exhibit the highest uncertainty range (−98.36% to +520.7%), followed by hogs and work cattle, reflecting the challenges in quantifying emissions from intensive and diverse production systems. In contrast, emissions from cattle, though still uncertain, show comparatively narrower bounds (−86.32% to +137.26%). These wide ranges underscore the necessity for improved data collection, enhanced field-based measurements, and the development of livestock species-specific emission factors tailored to local conditions.

### 3.5. Driving Forces and Policy Implications

**Figure 5:**
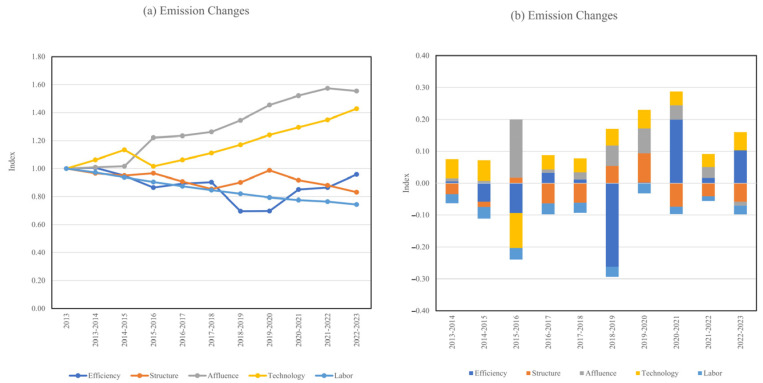
Time series decomposition multiplicative (**a**) and additive (**b**) decomposition of GWP emissions in agriculture livestock at the national level.

The time series decomposition of agricultural livestock GWP emissions at the national level is presented in Figure 5, which includes both multiplicative (Figure 5a) and additive (Figure 5b) forms.

Figure 5a illustrates the long-term evolution of emission-driving factors in China’s agricultural sector from 2013 to 2023. Among the five contributors, affluence exhibited the most pronounced upward trend, rising steadily from the baseline to nearly 1.6 in 2023. This indicates that the improvement of living standards and increasing demand for animal products have been the dominant drivers of emission growth. Technology also showed a consistent increase, reaching over 1.4 by 2023, suggesting that the intensification and modernization of livestock production have exerted additional upward pressure on emissions. In contrast, efficiency declined markedly after 2013, reaching a minimum of ~0.7 in 2019–2020, before recovering slightly. This pattern demonstrates that efficiency improvements have played a significant role in mitigating emissions, although their effect has fluctuated. Structural changes presented a mild downward trend overall, contributing to emission reduction, but with intermittent rebounds during 2017–2020. Meanwhile, labor consistently decreased, reflecting rural depopulation and mechanization, which exerted a sustained but relatively modest mitigation effect.

The annual decomposition results (Figure 5b) further reveal the marginal contributions of each factor. Affluence and technology contributed positively in nearly all years, confirming their long-term role in driving emission increases. By contrast, efficiency exerted a strong negative effect in several years, particularly in 2015–2016 and 2018–2019 (−0.25 to −0.30), offsetting part of the growth driven by affluence and technology. However, efficiency temporarily turned positive in 2020–2021, weakening its mitigation role. Structural effects were relatively minor but variable, alternating between weak inhibition and promotion of emissions, with a noticeable positive effect in 2020–2021. Labor changes consistently contributed to emission reductions, though at a smaller magnitude compared to efficiency.

Overall, these results highlight a persistent tension between economic and technological growth versus efficiency and demographic shifts. While affluence and technology have continuously amplified agricultural emissions, improvements in production efficiency, structural optimization, and labor transitions have partially counteracted these pressures [43]. Nevertheless, the compensatory effects have proven insufficient to fully offset the increasing demand-driven emissions, indicating that without stronger technological innovation oriented towards mitigation [44], emissions from China’s agricultural sector are likely to remain on an upward trajectory.

The decomposition results provide critical insights for designing effective mitigation strategies in China’s agricultural livestock sector. First, the consistently positive contribution of affluence highlights the challenge of reconciling emission control with rising demand for livestock products. This underscores the need to promote dietary transitions and encourage the consumption of low-emission protein alternatives. Second, the persistent upward pressure from technology suggests that modernization, if oriented solely toward production intensification, may inadvertently accelerate emissions. Therefore, policy incentives should prioritize green technological innovation, including precision feeding, manure management, and low-emission breeding systems, to ensure that technological progress translates into emission reduction rather than expansion.

Meanwhile, the negative effects of efficiency and labor factors demonstrate the potential for emission abatement through improved production efficiency and structural transformation. Enhancing productivity per unit of input, together with promoting region-specific structural optimization—such as shifting production away from environmentally sensitive regions—could generate substantial co-benefits for both climate and air quality. Furthermore, the observed variability of structural effects suggests that regionalized and species-specific policies are essential, targeting dominant sources such as pigs in eastern and southwestern China.

In sum, the findings indicate that China’s agricultural emission mitigation should rely on a dual approach: curbing demand-side growth while accelerating efficiency-oriented and low-carbon technological innovations. Such integrated strategies would enable the sector to balance food security with emission reduction, thereby contributing to national carbon neutrality and global climate goals.

### 3.6. Provincial Discrepancies and Mitigation Strategies

Provincial livestock manure GWP emissions in China between 2013 and 2023 exhibited substantial spatial heterogeneity, with both increases and declines observed across provinces (Table 4). The regional heterogeneity of livestock manure GHG emissions suggests that uniform mitigation strategies may not be effective. Instead, region-specific policies are needed. Sichuan, Henan, and Shandong consistently ranked among the largest contributors, reflecting their intensive livestock production bases and high animal population densities. Specifically, Sichuan maintained the highest emissions until 2020, after which Shandong surpassed it, reaching 24.48 Tg in 2023, while Henan shifted from the second-largest emitter in 2013–2017 to third place by 2023. Provinces in central and southern China, such as Hunan (18.39 Tg in 2023), Guangxi (16.46 Tg), and Guangdong (16.55 Tg), also remained within the top ten, underscoring their persistent importance in the national livestock emission profile. These high-emission provinces (Sichuan, Shandong, Henan, Hunan, Guangdong, Guangxi) should be prioritized for targeted manure management interventions, including improved biogas recovery, composting, and precision feeding technologies. Given their scale, even modest reductions could deliver substantial national benefits.

By contrast, metropolitan regions with limited livestock activity—including Beijing, Shanghai, and Tianjin—consistently reported the lowest emissions (<1 Tg) and remained at the bottom of the national ranking (28th–31st). Western provinces such as Tibet, Qinghai, Ningxia, and Hainan also showed relatively low emissions (<3 Tg), largely due to smaller populations and reduced demand for intensive livestock production. The contribution of low-emission regions (Beijing, Shanghai, Tianjin, Tibet, Qinghai, Ningxia, Hainan) to national totals is limited, and they can serve as pilot zones for innovative manure treatment technologies and provide replicable models for sustainable urban–rural integration in livestock management.

However, pastoral regions like Inner Mongolia and Xinjiang contributed moderate but non-negligible amounts, ranging from 7 to 10 Tg in recent years; although emissions are moderate, these areas host extensive ruminant systems with strong cultural and livelihood importance. Policies should focus on promoting grazing management, feed efficiency, and ruminant-specific mitigation options (e.g., methane inhibitors). Emerging hotspots (Anhui, Fujian, Jiangxi, Yunnan) experienced accelerated emission growth after 2017, indicating shifts in production patterns. Early implementation of mitigation measures in these areas can prevent the entrenchment of high-emission production systems.

## 4. Conclusions and Limitations

This study provides one of the first decadal-scale assessments of livestock-related NH_3_, N_2_O, and CH_4_ emissions in China during 2013–2023, highlighting both persistent and shifting emission patterns. Our results show that national NH_3_ emissions declined slightly (−3.7%), whereas N_2_O (+9.5%) and CH_4_ (+35%) increased, leading to a 24% rise in the aggregated GWP (from ~1100 Tg to ~1370 Tg CO_2_-eq). Swine remained the dominant contributors to NH_3_ and N_2_O, while poultry emissions also rose significantly in recent years, reflecting intensified production. Spatially, the North China Plain and the Sichuan Basin persisted as major hotspots, while new high-emission regions emerged in Hunan, Jiangxi, Heilongjiang, and Xinjiang after 2017, indicating structural shifts in livestock production. Despite only modest national-scale changes, the analysis revealed pronounced source- and region-specific heterogeneity, underscoring the necessity of differentiated mitigation approaches. High-emission provinces should be prioritized for intensive manure management interventions, pastoral regions require ruminant-specific measures, low-emission areas may serve as innovation pilots, and emerging hotspots warrant early preventive action. Taken together, these findings demonstrate that uniform strategies are unlikely to be effective, and that region- and species-targeted mitigation pathways are essential to simultaneously advance air quality improvement and support China’s national climate goals.

Although this study updates 2013–2023 livestock manure emission trends and couples them with key drivers, providing recommendations for emission reductions, two limitations still need to be improved in future studies. First, the driver analysis considers only macroeconomic development levels, overlooking potential trade-offs, co-benefits, and implementation barriers, as well as explicit policy interventions. Incorporating policy-specific scenarios and quantifying their impacts on future emission trajectories would enhance the study’s depth and policy relevance. Second, future work should model how livestock NH_3_ emissions and their mitigation alter particulate-matter composition and how greenhouse gas emissions and mitigation modify radiative forcing. The implications for carbon neutrality and air quality improvement should be articulated more explicitly.

## Figures and Tables

**Table 1:** Emission source categorization in the livestock sector and data sources.

Category	Sub-Category	Category	Sub-Category
Livestock ^a^	Cow	Poultry ^b,c^	Rabbit
	Beef cattle		Broiler
	Sow		Meat duck
	Hog		Meat goose
	Mutton		Laying goose
	Goat		Laying hens
	Mule		Laying duck
	Donkey		
	Horse		
	Work cattle		

^a^ Data collected from China Statistical Yearbook, 2014–2024 [3]. ^b^ Data collected from the Compilation of Statistics Data of Chinese agriculture 2014–2024 [35]. ^c^ Data collected from the China Rural Statistical Yearbook 2014–2024 [5].

**Table 2:** Descriptions of variables referred to in this paper.

	Driver	Variables	Symbol	Unit
*∆α*	Production efficiency	Livestock emissions	E	Tg
*∆β*	Industry structure	Livestock gross domestic product	S	Yuan
*∆γ*	Affluence	Agricultural gross domestic product	A	Yuan
*∆ρ*	Technology	Total power of agricultural machinery	M	Kw
*∆δ*	Labor force	The rural population	L	Person
		The total population	P	Person

**Table 3:** Uncertainty analysis (95% confidence level).

Category	Sub-Category	NH_3_	N_2_O	CH_4_
Livestock	Cow	(−92.65%; 185.47%)	(−68%; 75.06%)	(−80.16%; 109.54%)
	Beef cattle	(−83.4%; 156.59%)	(−55.99%; 52.97%)	(−67.72%; 139.75%)
	Sow	(−90.48%; 314.47%)	(−30.63%; 39.67%)	(−74.53%; 91.54%)
	Hog			
	Mutton	(−81.84%; 220.05%)	(−82.88%; 230.43%)	(−58.48%; 83.31%)
	Goat			
	Mule	(−90.39%; 175.22%)	(−62.34%; 63.68%)	(−36.64%; 28.63%)
	Donkey			
	Horse			
	Work cattle	(−82.73%; 119.03%)	(−66.18%; 70.92%)	(−23.65%; 16.7%)
Poultry	Rabbit	(−57.69%; 55.81%)	(−2.3%, 2.34%)	(−53.87%, 49.76%)
	Broiler			
	Meat duck			
	Meat goose			
	Laying goose			
	Laying hens			
	Laying duck			
Total		(−70.51%; 112.77%)	(−52.34%; 71.63%)	(−67%; 136.07%)

**Table 4:** Livestock GWP emissions and ranks at provincial level.

	Total Carbon Emissions (Tg)				Rank			
Regions	2013	2017	2020	2023	2013	2017	2020	2023
Beijing	0.77	0.54	0.27	0.18	30	30	31	31
Tianjin	0.90	0.73	0.83	0.81	28	29	29	29
Hebei	10.52	10.11	11.57	13.75	7	7	7	8
Shanxi	2.54	2.63	3.13	4.62	23	22	22	22
Inner Mongolia	8.27	7.73	9.07	10.22	10	12	12	12
Liaoning	7.52	6.49	9.71	11.59	11	14	10	11
Jilin	4.87	4.44	5.60	7.14	19	19	18	17
Heilongjiang	6.00	6.07	6.62	8.12	15	15	16	16
Shanghai	0.56	0.45	0.36	0.37	31	31	30	30
Jiangsu	7.37	6.60	8.09	9.63	13	13	13	15
Zhejiang	3.95	2.26	2.40	3.28	21	23	24	23
Anhui	7.44	6.93	9.54	13.20	12	12	11	9
Fujian	5.28	4.63	6.58	10.21	16	18	16	13
Jiangxi	7.24	7.00	7.77	10.18	14	11	14	14
Shandong	14.87	14.71	18.20	24.48	3	3	2	1
Henan	17.54	15.23	17.20	20.81	2	2	3	2
Hubei	10.82	10.86	10.18	14.33	6	5	9	7
Hunan	13.85	13.99	14.24	18.39	4	4	4	4
Guangdong	10.29	9.64	11.92	16.55	8	8	6	5
Guangxi	11.84	10.58	12.50	16.46	5	6	5	6
Hainan	1.96	1.60	1.70	2.30	26	26	26	26
Chongqing	4.51	3.81	4.49	5.48	20	20	20	20
Sichuan	18.58	16.74	18.74	20.53	1	1	1	3
Guizhou	5.16	4.68	4.89	5.73	18	17	19	19
Yunnan	8.21	8.98	10.47	13.20	10	9	8	10
Tibet	2.33	2.03	1.87	2.62	24	25	25	25
Shaanxi	2.31	2.22	2.49	2.95	25	24	23	24
Gansu	2.72	2.66	3.45	4.64	22	21	21	21
Qinghai	1.51	1.45	1.67	1.77	27	27	27	27
Ningxia	0.87	0.94	1.25	1.70	29	28	28	28
Xinjiang	5.26	5.33	5.69	6.93	17	16	17	18

## Data Availability

The original contributions presented in the study are included in the article/Supplementary Material; further inquiries can be directed to the corresponding authors.

## Supplementary Materials

The following supporting information can be downloaded at https://www.mdpi.com/article/10.3390/toxics13110933/s1. Table S1: The number of livestock and poultry breeding in 2023 (ten thousand heads). Table S2: NH_3_ emission factors from livestock manure management. Table S3: N_2_O emission factors from livestock manure management (kg N_2_O/head). Table S4: CH_4_ emission factors from livestock manure management (kgCH_4_/head). Table S5: Parameters used for uncertainty analysis.
